# Evidence for long memory in focal seizure duration

**DOI:** 10.1002/epi4.12457

**Published:** 2021-01-07

**Authors:** Joline M. Fan, Sharon Chiang, Vikram R. Rao

**Affiliations:** ^1^ Department of Neurology and Weill Institute for Neurosciences University of California, San Francisco San Francisco CA USA

**Keywords:** capacitative effect, chronic EEG, interseizure interval, responsive neurostimulation

## Abstract

**Objective:**

A major source of disability for people with epilepsy involves uncertainty surrounding seizure timing and severity. Although patients often report that long seizure‐free intervals are followed by more severe seizures, there is little experimental evidence supporting this observation. Optimal characterization of seizure severity is debated; however, seizure duration is associated with seizure type and can be quantified in electrographic recordings as a limited proxy of clinical seizure severity. Here, using chronic intracranial electroencephalography (cEEG), we investigate the relationship between interseizure interval (ISI) and duration of the subsequent seizure.

**Methods:**

We performed a retrospective analysis of 14 subjects implanted with a responsive neurostimulation device (RNS System) that provides cEEG, including timestamps of electrographic seizures. We determined seizure durations for isolated seizures and for representative seizures from clusters determined through unsupervised methods. For each subject, the median ISI preceding long‐duration seizures, defined as the top quintile of seizure durations, was compared with the median ISI preceding seizures with durations in the residual quintiles. In a group analysis, the mean seizure duration and the proportion of long‐duration seizures were compared across ISI categories representing different lengths.

**Results:**

For 5 out of 14 subjects (36%), the median ISI preceding long‐duration seizures was significantly greater than the median ISI preceding shorter‐duration seizures. In the group analysis, when ISI was categorized by length, the proportion of long‐duration seizures within the high ISI category was significantly higher than that of the low ISI category (*P* < 0.001).

**Significance:**

By leveraging cEEG and accounting for seizure clusters, we found that the likelihood of long‐duration seizures positively correlates with ISI length, in a subset of individuals. These findings corroborate anecdotal clinical observations and support the existence of capacitor‐like long memory processes governing the dynamics of focal seizures.

**Key Points**

- Chronic intracranial EEG enables investigation of how the interval between seizures relates to seizure duration
- Five of fourteen subjects were identified to have an association between long ISI and likelihood of long‐duration seizures
- The likelihood of long‐duration seizures, as compared to the standard measure of seizure duration, may offer an alternative measure of seizure severity that increases with ISI in a subset of subjects
- Findings corroborate anecdotal clinical observations and support the concept of a “capacitative effect” underlying the dynamics of focal seizures

## 1 INTRODUCTION

Seizure unpredictability is one of the most disabling factors in epilepsy.^1^ The unpredictability relates not only to seizure timing, but also to seizure severity.^2^, ^3^, ^4^ For example, a single prolonged convulsive seizure may cause greater morbidity than a cluster of brief focal aware seizures. Although methods to predict seizure timing are emerging,^5^ methods to anticipate seizure severity are still lacking.^6^, ^7^

A common clinical observation made by patients is that longer periods of seizure freedom appear to give rise to more severe seizures.^6^ If time since last seizure indeed informs the severity of future seizures, seizure forecasting models could potentially provide information about the likely morbidity of future seizures. However, experimental evidence to support this observation has been limited due to the need for accurate, quantitative metrics of seizure severity. Although multiple indices have been developed to capture seizure burden aggregated over time,^2^, ^8^, ^9^, ^10^, ^11^ quantification of *individual* seizure severity remains challenging. Seizure duration has been demonstrated to relate to seizure type^12^, ^13^, ^14^, ^15^, ^16^—focal aware, focal impaired awareness, or focal to bilateral tonic‐clonic—and to seizure clusters.^17^ Given these associations, seizure duration may provide a glimpse into the underlying physiology and severity of individual seizures, and, importantly, can be quantitatively probed through chronic recordings.

Implantable devices, such as the RNS^®^ System, have now provided unique opportunities to continuously monitor cEEG in patients over many years. By capturing and recording key metrics of the cEEG, the RNS System has revealed many insights about seizure dynamics, including cycles of seizure risk,^18^ medication effects,^19^ and seizure lateralization and localization.^20^, ^21^, ^22^

In this study, we utilize the RNS System to characterize the relationship between ISI and seizure duration. Initial studies utilizing the NeuroVista dataset to investigate the relationship between seizure duration and ISI^6^, ^23^ demonstrated distinct groupings of seizure duration and ISI in some subjects; in two of fifteen subjects, short‐duration seizures were statistically associated with short ISI.^6^ Other studies, including in canine epilepsy models,^24^ suggest that the relationship between preceding ISI and seizure duration varies across subjects. This variability may be related to the effects of seizure clusters, which were not accounted for in these analyses, and may obscure the extent to which seizure duration demonstrates “memory” of prior events. Here, we sought to clarify the relationship between preceding ISI and seizure duration by (a) leveraging a distinct dataset involving long‐term cEEG from the RNS System, (b) accounting for seizure clusters, and (c) providing key additional metrics of long‐duration seizure likelihood. Based on clinical observations, we hypothesized that ISI length predicts seizure duration.

## 2 METHODS

### 2.1 Subject selection

Forty‐five subjects followed at the University of California, San Francisco (UCSF) Medical Center for medically refractory focal epilepsy and implanted with an RNS System for purely clinical indications between 8/2014 and 2/2018 were considered for this study. Data collection was approved by the IRB at UCSF, and written informed consent was obtained from all subjects.

### 2.2 Data collection

Timestamps and durations of long episodes (LE), sustained detections of epileptiform activity exceeding a prespecified threshold duration, were obtained from NeuroPace, Inc To determine the extent to which LE represented electrographic seizures, visual inspection of RNS System electrocorticograms (ECoGs) containing LE was performed by an experienced epileptologist (VRR), as described previously.^18^ During each epoch of stable device detection settings, all stored ECoGs containing LE were reviewed.^18^ Twenty‐five subjects were identified for whom LE was a reliable indicator of an electrographic seizure with a positive predictive value (PPV) greater than 75%. Subjects with fewer than 15 isolated seizures or clusters, or a seizure frequency greater than 1 per day, were excluded due to limited variability in ISI length, leaving a total of 14 subjects included in this study. LE timestamps and durations were obtained from the longest period of stable detection settings for each subject. Identification of a period with stable detection settings is an essential step, in order to avoid variability arising solely from changes in the threshold or detection criteria of epileptiform activity.

### 2.3 Data processing

MATLAB R2019a was used for analysis. Seizure clusters were defined using unsupervised temporal clustering via change‐point analysis with bootstrapping. This approach allows for unsupervised seizure cluster detection while enabling for adjustments to satisfy clinical criteria.^25^ The change‐point threshold is a manually adjusted parameter that accounts for differences in seizure frequency affecting the definition of meaningful seizure clusters. The change‐point threshold was titrated for each subject based on two criteria: (a) The maximum intracluster ISI was between 6 and 24 hours, and (b) the mean intracluster ISI was closest to 1.5 hours without exceeding 1.5 hours, depending on the direction of titration (ie, if the mean intracluster ISI was below 1.5 hours, the value was increased to be the maximal value less than 1.5 hours, and vice versa if above 1.5 hours). Although there is currently no standardized method to adjust the change‐point threshold, this titration methodology enabled individualized cluster identification such that lower thresholds were used for subjects with near‐daily seizures and higher thresholds were used for subjects with more sparse seizures. All identified seizure clusters were manually reviewed (JMF). The average intracluster ISI ranged from 1.11 to 2.78 hours, with the exception of Subject 8, who required a longer intracluster ISI to capture clusters due to a low number of LEs (<5) involved in seizure clusters with relatively higher intracluster ISIs (Table 2, Figure S1).

For each isolated seizure or initial seizure of a cluster, the ISI was calculated as the duration of time between the seizure and the most recent isolated seizure or end of a seizure cluster. To compare across subjects in the group analysis, ISIs were linearly scaled to a standardized value from 0 to 100. The linear multiplier was determined by dividing 100 by the maximum ISI for each subject. For seizure clusters, the representative duration of the seizure cluster was set to the maximum duration seizure within the seizure cluster and the time of the cluster was set to the time of the first seizure within the cluster.

### 2.4 Statistics

Two separate methods were used to evaluate the association between ISI and seizure duration for individual subjects. First, for each subject, the Wilcoxon test was used to evaluate for a significant difference in ISI length between long‐duration seizures, defined as seizures in the top quintile of seizure durations, versus shorter‐duration seizures, defined as those in the residual quintiles (bottom 80%) of seizure durations. Statistical significance was determined with false discovery rate (FDR) control of 0.15. Second, kmeans++ was used to cluster scaled ISI lengths into *K* = 3 groups corresponding to “low,” “medium,” and “high” ISI lengths. Cluster centroids were initialized using the 25th, 50th, and 75th percentiles of ISIs and transformed by the natural log to enable sufficient sampling for all ISI groupings. Resultant “low,” “medium,” and “high” ISI groups are shown in Figure S2. Sensitivity of kmeans++ groupings to *K* = 2 and *K* = 4 clusters was additionally performed. One‐way ANOVA was used to evaluate for a significant difference in the proportion of long‐duration seizures and mean seizure duration between ISI categories. Post hoc pairwise statistical significance was corrected for multiple comparisons with an FDR of 0.05.

## 3 RESULTS

### 3.1 Demographic characteristics and quantitative electrographic metrics

Table 1 shows the demographic and RNS System characteristics for the study sample. The average age of subjects implanted was 41.3 ± 15.1 years. The mean number of days with stable detection settings was 710 ± 276 days (range, 287‐1385 days). Subjects represented a wide range of RNS System lead locations, including bilateral hippocampal, neocortical, and mixed (mesial temporal and neocortical). Six subjects had focal aware seizures (FAS) or focal impaired awareness seizures (FIAS) without secondary generalization, and nine subjects had FAS and/or FIAS with focal to bilateral tonic‐clonic seizures (FBTCS).

**TABLE 1:** Demographics, characteristics of the RNS System, and clinical seizure types

Subject	Age (RNS placed)	Gender	Days recorded	LE duration^a^ (s)	Avg LE frequency (LE/m)	Lead location	Clinical seizure type		
							FAS	FIAS	FBTCS
1	46	M	588	30	5.98	Bilateral hippocampal		x	
2	69	F	698	30	28.63	Cingulate, hippocampus		x	
3	44	M	287	30	26.81	Bilateral hippocampal		x	x
4	32	F	664	15	6.78	Lateral occipital	x		x
5	43	M	607	20	17.02	Motor strip	x		
6	39	F	642	30	28.44	Bilateral hippocampal		x	x
7	38	M	517	30	6.10	Bilateral hippocampal		x	x
8	41	M	1385	30	1.09	Lateral temporal, hippocampus		x	x
9	20	F	1062	30	2.71	Left inferior frontal (Broca's)	x		x
10	51	M	603	40	6.38	Bilateral hippocampal		x	
11	21	M	537	15	24.28	Orbitofrontal, hippocampus		x	
12	69	F	1055	30	1.81	Bilateral hippocampal		x	
13	42	F	664	30	19.44	Motor strip		x	x
14	23	F	642	30	7.60	Bilateral hippocampal	x	x	x

Abbreviations: F, female; FAS, focal aware seizures; FBTCS, focal to bilateral tonic‐clonic seizures; FIAS, focal impaired awareness seizures; LE, long episode; M, male.

^a^ RNS System parameter denoting the minimum duration of continuously detected epileptiform activity required to constitute a LE.

Table 2 shows characteristics of identified electrographic seizure clusters, including the mean number of electrographic seizures (LE) per cluster, mean inter‐ and intracluster ISI, maximum intracluster ISI, and average duration of seizure clusters. The mean number of seizures per cluster was 3.0 ± 3.8 seizures (range 1‐15; Table 2a). The average intercluster ISI, that is, ISI between isolated seizures or seizure clusters, ranged from 2 to 32 days, reflecting wide variability in seizure frequency from near‐daily to monthly seizures (Table 2b). The mean intracluster ISI was <3 hours (except for subject 8, see Methods; Figure S1) with a maximum intracluster ISI between 6.4 and 22.4 hours (Table 2d). The average of all maximum duration seizures within each cluster and isolated seizures is shown in Table 2e. Consistent with prior studies,^12^, ^13^, ^14^ subjects with FAS, for example, Subjects 4 and 5, tended to have short‐duration seizures, whereas those subjects with FBTCS (Subjects 3, 4, 6‐9, 13, and 14) tended to have long‐duration seizures.

**TABLE 2:** Quantitative electrographic metrics on cluster size, ISI, and duration

Patient	(a) Avg # LEs per cluster^a^		(b) Avg intercluster ISI (d)^b^		(c) Avg intracluster ISI (h)		(d) Max intracluster ISI (h)	(e) Avg duration (s)^c^	
	Mean	SD	Mean	SD	Mean	SD		Mean	SD
1	2.95	4.05	14.76	11.84	1.18	2.12	14.51	47.73	16.22
2	1.99	2.08	2.03	2.07	1.67	1.60	8.90	50.73	9.76
3	4.73	7.53	5.37	4.19	1.11	3.75	22.36	54.14	22.38
4	1.46	1.14	7.00	11.50	1.89	2.01	6.98	20.16	11.87
5	1.76	1.13	3.06	3.09	2.78	1.50	6.44	41.76	14.93
6	2.83	2.48	2.92	3.62	1.59	2.26	10.33	64.14	28.56
7	1.43	0.98	7.24	7.07	1.71	2.36	8.06	52.68	37.98
8	1.16	0.47	32.09	20.31	8.46	5.12	13.19	89.60	30.26
9	1.49	1.12	16.75	14.74	2.33	2.60	12.77	56.20	24.94
10	1.45	2.52	7.08	8.15	1.67	2.82	9.23	50.02	8.84
11	15.88	18.18	18.48	19.28	1.55	3.28	18.20	37.45	10.64
12	1.21	0.54	21.34	25.41	2.33	2.44	6.45	47.07	13.50
13	2.05	1.45	3.12	2.69	1.63	3.67	19.79	39.53	6.08
14	1.09	0.30	4.52	4.86	2.45	3.01	7.25	63.74	25.55

Note

Mean and standard deviation (SD) are provided.

^a^ Including isolated seizures.

^b^ Between isolated seizures or clusters.

^c^ Averaged max duration.

### 3.2 Derivation of metrics on example subject

**FIGURE 1:**
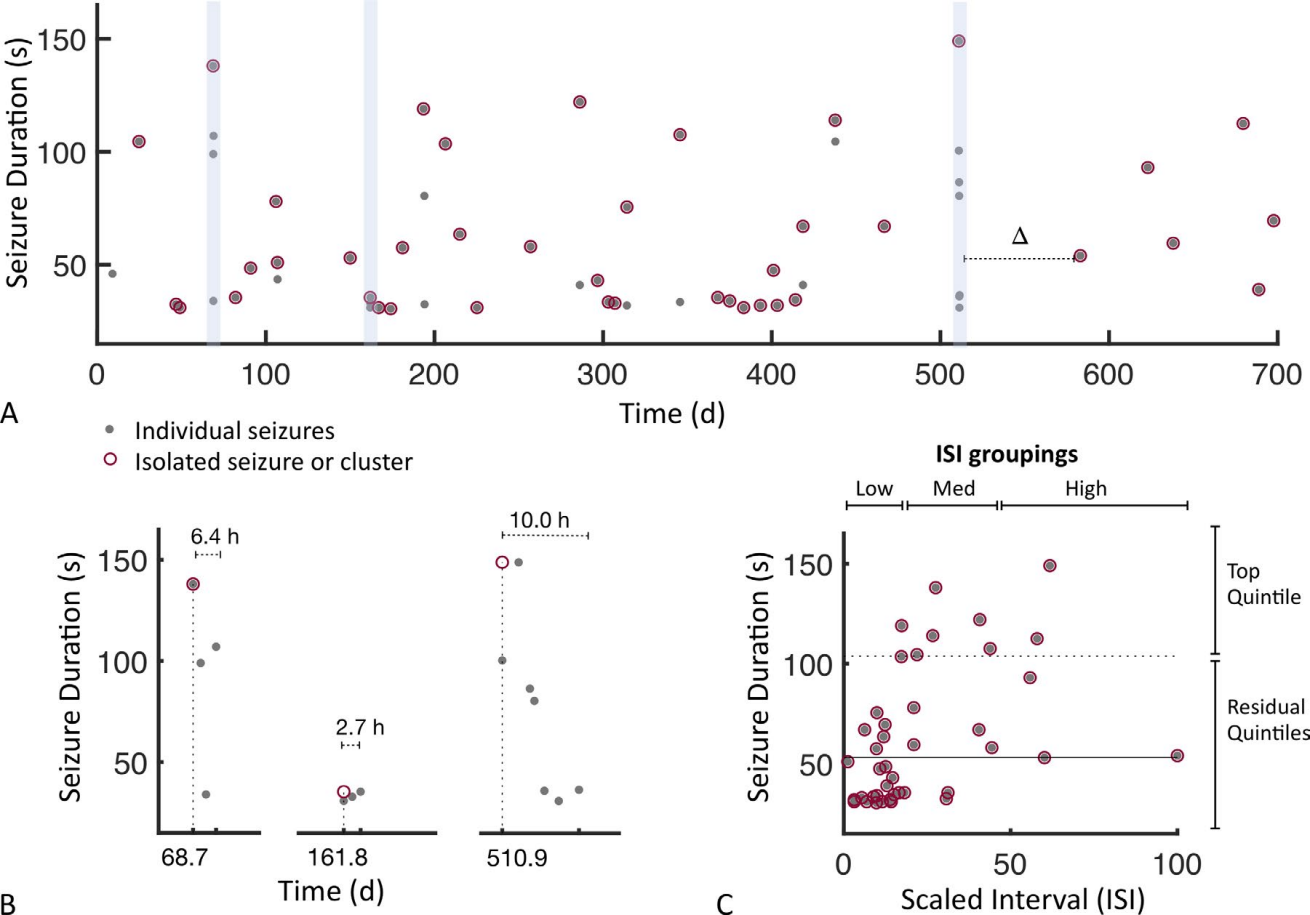
Seizure durations and interseizure intervals. A, Data from an example subject (Subject 9). Gray dots, electrographic seizures; red circles, isolated seizures or seizures of maximal duration within identified seizure clusters; delta (Δ), ISI corresponding to the isolated seizure occurring just after the dotted line. Blue shading highlights seizure clusters, expanded in (B). B, In the three seizure clusters shaded in (A), red circles denote representative seizure cluster parameters, illustrating the maximum duration and seizure onset time (dotted line) of the initial seizure within a cluster. C, Durations of isolated seizures and representative seizures from clusters versus corresponding scaled ISI. Solid line, median seizure duration; dotted line, top quintile. Top, ISI groupings based on length; right, top and residual quintiles of seizure durations

Seizure time series in individual subjects revealed that seizures occur as isolated events and as clusters (Figure 1A). In contrast to prior observations of clinical seizure clusters,^17^ temporal clusters identified from unsupervised techniques (see Methods) revealed that the seizure with maximum duration within a cluster was not necessarily the terminal event. Across subjects, the percentage of seizure clusters for which the maximal duration seizure fell on the terminal seizure of the cluster was 42 ± 18% (range 15%–83%). In order to account for the variable positioning of the maximum duration seizure within a cluster, a seizure cluster was represented by the onset time of the first electrographic seizure and the maximum seizure duration within the cluster (Figure 1B, red circles). By accounting for both isolated seizures and seizure clusters, a positive association between the ISI and seizure duration is shown in an example patient (Figure 1C). Figure 1C further provides an illustration of the ISI groupings by length and the threshold used for long‐duration seizures, defined as the top quintile of seizure durations.

### 3.3 Comparison of ISI lengths between longer‐ and shorter‐duration seizures

**FIGURE 2:**
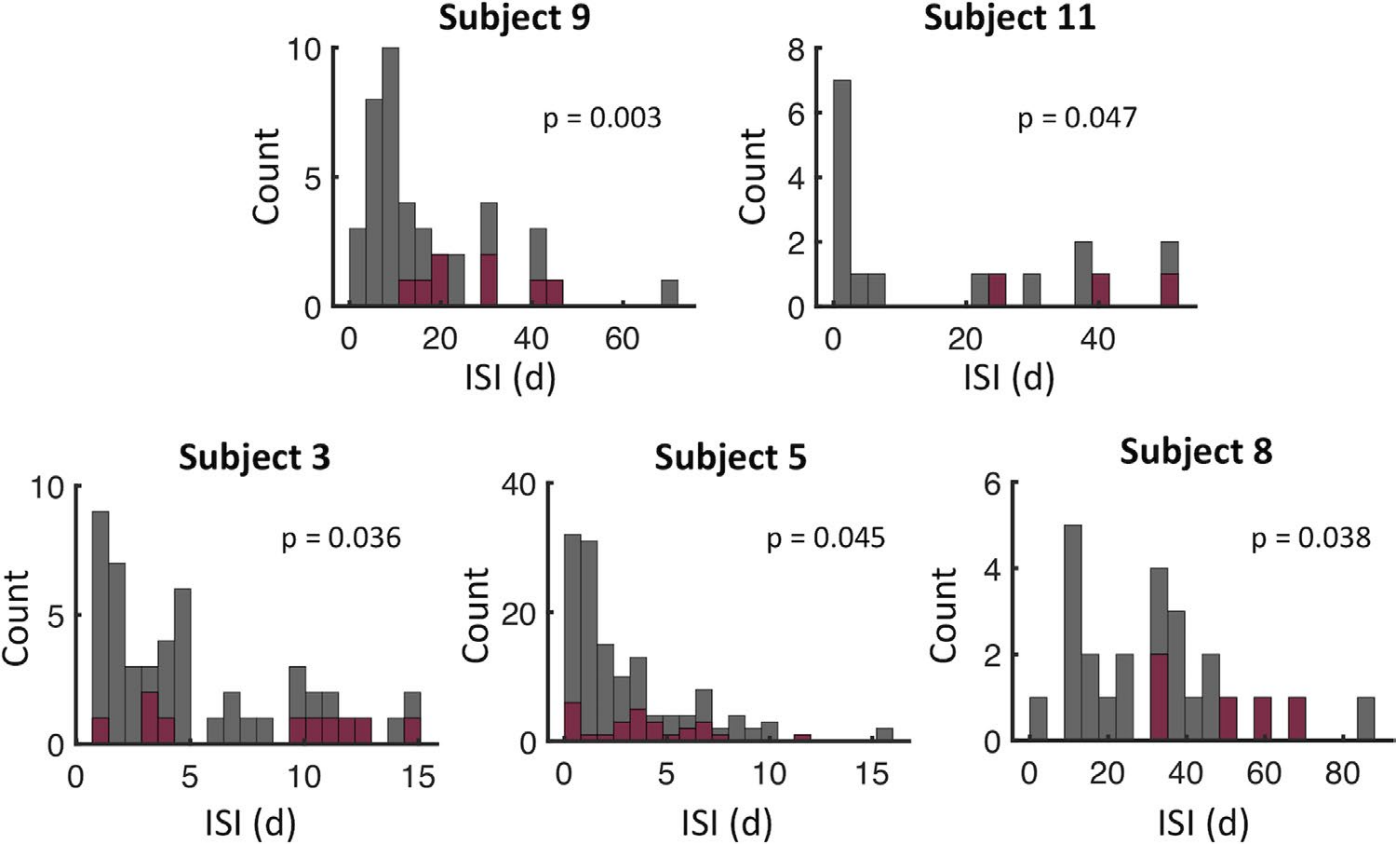
Distribution of ISI corresponding to long‐ and shorter‐duration seizures. Distributions of ISI for long‐duration (red bars, top quintile) and shorter‐duration (gray bars, residual distribution) seizures for the subgroup of five subjects with significant difference between the two

In order to determine whether long‐duration seizures were associated with longer ISI, the distribution of ISIs preceding long‐duration seizures was compared to that of shorter‐duration seizures, that is, the residual distribution, for each subject. As seen in Figure 2, in five of fourteen subjects (36%), the median ISIs preceding long‐duration seizures were significantly longer than the median ISIs preceding shorter‐duration seizures by an average of 17.6 ± 14.8 days. The distribution, mean, and median representing all subjects are illustrated in Figure S3 and Table S1.

### 3.4 Group comparison of seizure durations for high, medium, and low ISIs

**FIGURE 3:**
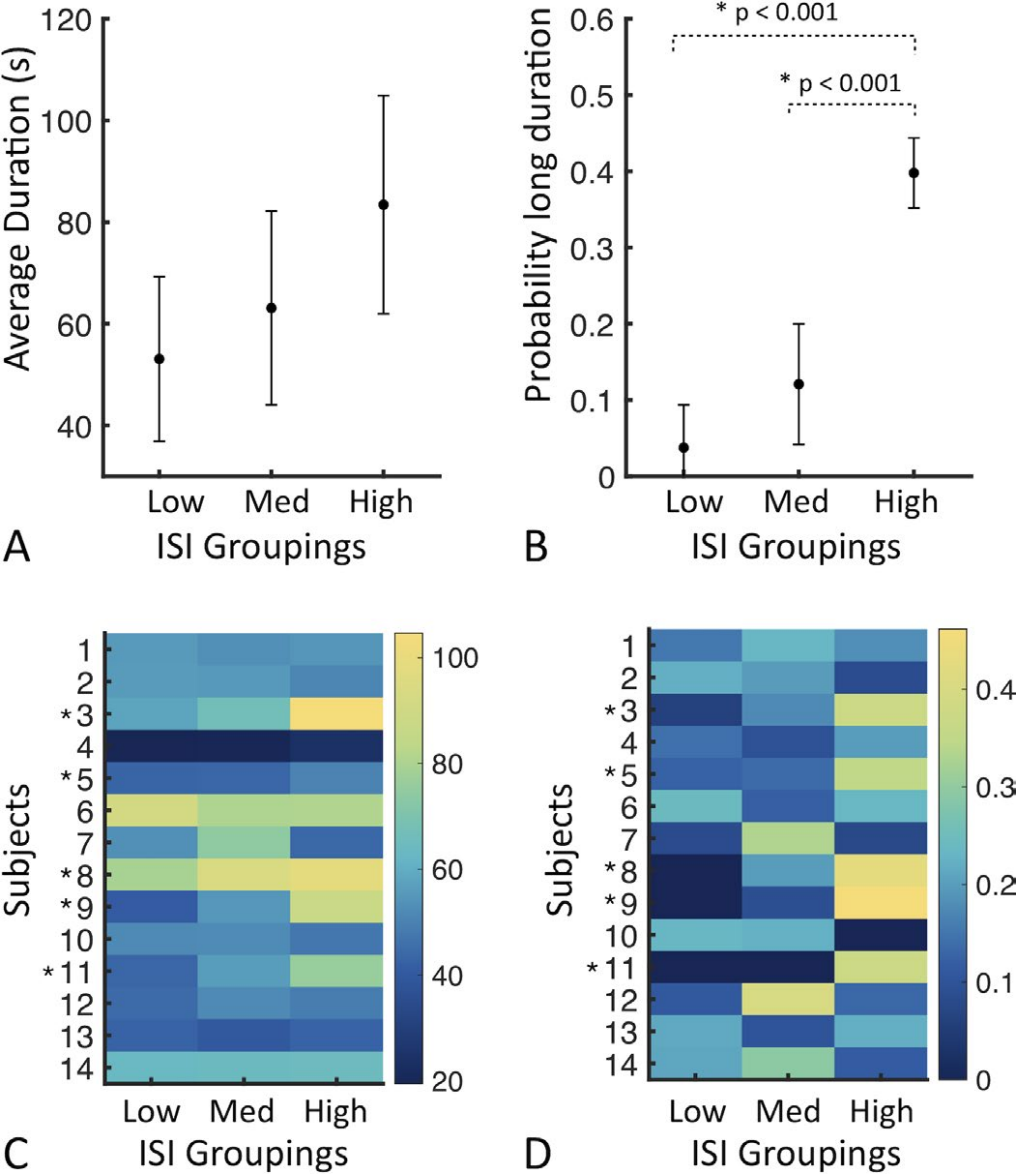
High ISI increases probability of long‐duration seizures in a subset of subjects. A, Average duration of seizures per ISI category in the subset of subjects shown in Figure 2. Error bars denote standard deviation. B, Proportion of long‐duration seizures (ie, top quintile of seizure durations) for each ISI category across the same subset of subjects. C, Visualization of the average seizure duration across all subjects. D. Visualization of the probability of long‐duration seizures across all subjects. Asterisks in (C) and (D) indicate subjects from Figure 2 whose data are analyzed in (A) and (B)

Next, in order to pursue a group analysis across patients with highly variable ISI distributions, the ISIs for each subject were linearly scaled to a standardized value (0‐100) and categorized by length into “low”, “medium,” and “high” ISI categories. Across the subset of subjects (5/14), for whom long ISI preceded long seizures, mean seizure duration was 53.1 ± 16.2 seconds, 63.19 ± 19.1 seconds, and 83.4 ± 21.5 seconds for the low, medium, and high ISI categories, respectively (Figure 3A). Seizure duration was not significantly different between ISI groupings (*P* = 0.072, one‐way ANOVA) (Figure 3A). Given that differences in the likelihood of long‐duration seizures might not be revealed by seizure duration alone due to its nonuniform distribution,^6^ we asked whether the proportion of long‐duration seizures varied across the ISI categories. Across the same subset of patients (5/14), a significant difference between proportion of long‐duration seizures and the three ISI groupings was identified (*P* < 0.001, one‐way ANOVA). Pairwise hypothesis testing revealed a significantly lower proportion of long‐duration seizures in low and medium ISI groupings, as compared to high ISI groupings (*P* < 0.001; Figure 3B). The difference in the proportion of long‐duration seizures was not statistically significant between the low ISI and the medium ISI categories (*P* = 0.12). Results were robust to *K* = 2 and *K* = 4 clusters (Figure S4).

In comparison, the mean seizure duration and the proportion of long‐duration seizures for “low,” “medium,” and “high” ISI groupings are shown for the overall group of 14 subjects in Figure 3C‐D. No significant difference in the average seizure duration (*P* = 0.591) or the proportion of long‐duration seizure (*P* = 0.064) was present between the three ISI groups in the total sample. These findings confirm that the association of high ISI with longer seizure durations is apparent only in a subset of subjects.

## 4 DISCUSSION

This study demonstrates that, in a subset of subjects, high ISI is associated with increased probability of long‐duration seizures. By employing chronic recordings from the RNS System and utilizing seizure duration as a proxy for seizure severity,^12^, ^14^ these findings corroborate anecdotal clinical observations that long periods of seizure freedom may give rise to more severe seizures in some individuals.

Five of fourteen subjects were identified to have high ISIs that corresponded to long‐duration seizures. Additionally, the proportion of long‐duration seizures was identified to be a more sensitive metric and increased with ISI, as compared to average seizure duration. Other studies^6^, ^24^, ^26^, ^27^ have utilized alternative approaches to studying the relationship between seizure duration and preceding ISI length. Supporting an association, stochastic models in rodents^27^ illustrated that seizure duration can be modeled as a function of the ISI, which was further demonstrated to be a function of the duration of a prior seizure. A complex relationship was observed between seizure duration and ISI in canine models, with one of six dogs demonstrating a positive correlation.^24^ While lead and clustered seizures were denoted in the study by Gregg et al, intercluster ISIs remained by definition associated with only the lead seizure.

Cook et al described the complex unimodal or bimodal distributions of electrographic seizures in human chronic recordings and identified two subjects in which short‐duration seizures were more likely associated with short ISI.^6^ The current study supports and advances the prior studies by accounting for seizure clusters, which would otherwise greatly change the interpretation of ISI with respect to individual events. For instance, if seizure clusters are not considered, a long‐duration intracluster seizure may be attributed to a short ISI, as measured within the cluster, rather than more appropriately measured from the prior cluster or isolated seizure remote from the current cluster. Furthermore, an intercluster ISI would be associated only with the lead seizure, rather than weighed appropriately to consider the entire seizure cluster. Without accounting for seizure clusters, an association between ISI and duration has a tendency be diluted.

In contrast to prior studies,^17^ we observed that the maximum duration seizure within a seizure cluster occurred at variable positions within a cluster, which is compatible with findings from other studies using chronic intracranial recordings.^6^, ^24^ Because of these observations, the maximum duration seizure within a cluster, rather than the lead or terminal seizure, was used to represent seizure duration for the cluster. The difference in these findings as compared to the self‐trigger hypothesis^17^ may relate to differences in clustering methods or environmental factors. Rather than an ambulatory setting, the study by Ferastraoaru et al^17^ was performed in an inpatient video‐EEG monitoring unit, where medications are frequently altered on a daily basis.

The probability of a long‐duration seizure was found to be an alternative and potentially more sensitive summary statistic for seizure severity. This finding supports a prior finding that the durations do not linearly increase,^6^ as seizure durations are not uniformly distributed, but rather are often multimodal^6^ and reflect discrete seizure types.^12^, ^13^, ^14^, ^15^, ^16^ Clustering ISI into two to four groups demonstrates that these findings are independent of the number of groupings. Given that longer‐duration seizures are associated with FBTCS, this finding suggests that longer ISIs may increase the likelihood of FBTCS. While seizure duration provides some insight into the seizure type,^12^, ^14^ the association of seizure type with ISI is not within the scope of study and would be of interest for further work. Importantly, the definition of long‐duration seizure was patient‐specific; as seen in Figure S2, the upper quintile was defined on an individual patient basis. In this sense, the relative metric speaks more directly to the clinical observation described by patients, who are most familiar with their own seizure dynamics. Although beyond the scope of this study, examining the correlation between patients’ perceptions of seizure severity and actual seizure durations, assessed using cEEG, would be of interest for future work. Additional analysis of the clinical phenotypes of the subset of patients for whom there was an association between ISI and long‐duration seizures did not reveal any obvious commonalities in demographic or RNS System characteristics, including lead location or seizure type.

The mechanism(s) underlying the relationship between ISI and seizure duration is unknown but may relate to the long‐term memory processes of an epileptic network.^26^ In animals and humans, seizures do not occur randomly; they have a tendency to cluster in time^28^, ^29^, ^30^, ^31^ and burst in cyclical rhythms,^18^, ^23^, ^32^, ^33^ which implies that the epileptic network has an inherent memory of prior events.^23^ Individual seizures and clusters have been associated with long periods of seizure freedom,^34^ which have been thought to relate to long durations of postictal suppression.^35^ Insufficient postictal inhibition^17^, ^36^ or excess excitation^37^ has also been implicated in seizure clusters. The mechanisms underlying the inhibitory and excitatory balance that drives seizure timing and inherent network memory are hypothesized to play a possible role in the “capacitative effect” seen here. Specifically, with longer periods of seizure freedom in an otherwise unchanged network, increased excitation that supersedes waning inhibition may in time lead to a network that sustains seizure propagation, thereby increasing the duration of the subsequent seizure.

This study has limitations. The RNS System is not a pure recording device; rather, it applies electrical stimulation in response to detections of epileptiform activity. The implications of the frequent stimulation remain unknown, but long‐term neuroplasticity effects are likely.^38^ For example, Subject 6 demonstrated progressive reduction in seizure frequency without any identifiable behavioral or pharmacological interventions (Figure S5), possibly reflecting neuromodulatory effects of chronic stimulation. As a result, longer ISIs tended to occur during later time periods for this subject. The high ISI grouping therefore ultimately reflects a period of time when the subject achieved improved seizure control. Improved seizure control may affect the data by increasing the tendency of higher ISIs and shorter seizure durations.

Another limitation involves the generalizability of these findings. Specifically, the cohort studied here with cEEG represents a selected subgroup of patients with medically refractory focal epilepsy. Patients with primary generalized epilepsies or medically controlled epilepsies, for instance, are not included in this study, and therefore, the extent to which similar dynamics occur in the broader population of people with epilepsy cannot be inferred. In order to address other aspects of seizure severity, such as patient perception, future studies could examine electronic seizure diaries. Of note, the main finding in this study—an association between high ISI and the likelihood of long‐duration seizures in a subset of patients—does not help predict the timing of seizures. Rather, these findings complement ongoing efforts on predicting seizure occurrence,^39^, ^40^ by providing information that may help anticipate other dimensions of seizure dynamics.

In addition, there are important limitations to using seizure duration as a proxy for seizure severity, which has many objective and subjective determinants, including seizure type, frequency, loss of awareness, and patient and/or observer percept.^2^, ^3^, ^8^, ^9^, ^10^, ^11^ Here, electrographic seizure duration, an objective measure, is used to probe one aspect of seizure severity. Additional considerations include the possibility that patients may become more complacent after longer periods of seizure freedom, leading to medication noncompliance or engagement in higher risk behaviors that may trigger more severe seizures. Although we focused on ISI as a possible predictor of seizure duration, other potential predictors that were not examined include the underlying state of the network^41^ and cycles of interictal activity,^18^ both of which impact seizure risk and thus may also impact seizure duration.

Given that unpredictability in epilepsy negatively affects quality of life, next‐generation seizure forecasting systems would ideally estimate both the timing and severity of upcoming seizures. Future neurostimulation devices that incorporate information about prior events could potentially optimize clinical response by providing time‐varying, risk‐stratified therapy tailored to intrinsic memory processes in brain networks.

## CONFLICT OF INTEREST

VRR has received honoraria from NeuroPace, Inc for consulting and speaking engagements. The authors declare no targeted funding or compensation from NeuroPace, Inc for this study. The remaining authors declare no competing financial interests.

## ETHICAL APPROVAL

We confirm that we have read the Journal's position on issues involved in ethical publication and affirm that this report is consistent with those guidelines.

## Supporting information

Supplementary Material
