# Supplementary material for: Evidence for long memory in focal seizure duration
Source: Epilepsia Open. 2021 Jan 7;6(1):140–8. doi: 10.1002/epi4.12457 (PMC7918332; doi:10.1002/epi4.12457)
Supplement: Supplementary file 1 — Supplementary Material [file EPI4-6-140-s001.pdf]

Supplementary Materials:

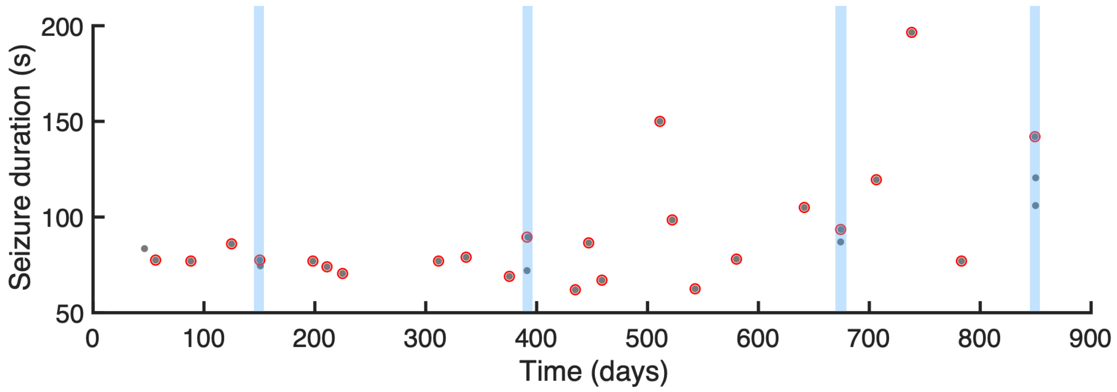

Figure S1. Demonstration of isolated seizures and seizure clusters for Subject 8. Due to the small number of seizure clusters, a higher mean intracluster ISI was required to achieved clusters. Across four intracluster ISIs, the mean ISI was 8.46 ± 5.12 h with a maximum ISI of 13.2 h.

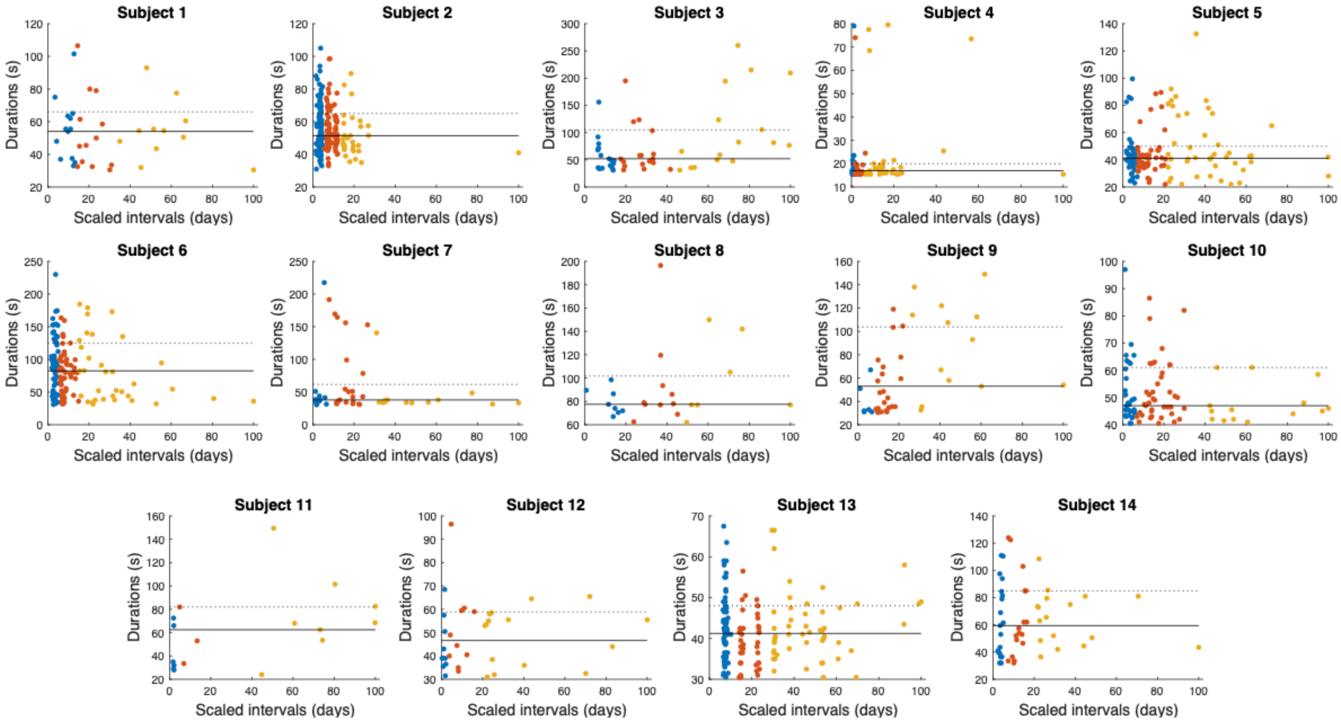

Figure S2. The relationship between maximal duration seizure and scaled ISI across 14 subjects included in this study. The points are color coded by the K-means++ division of "low" (blue), "medium" (red), and "high" (yellow) ISI groupings.

Table S1. ISI statistics of long-duration and shorter-duration seizures.
In five subjects (bold), median ISI of long-duration seizures was significantly larger than median ISI for shorter-duration seizures.

Subject	Median ISI of Long-duration† seizures (d)	Mean ISI of Long-duration† seizures (d)	Median ISI of Shorter-duration§ seizures (d)	Median ISI of Shorter-duration§ seizures (d)	ISIs of long duration seizures vs. Residual (p-value)*
1	10.8	14.1	9.4	14.9	0.985
2	1.0	1.6	1.8	2.1	0.070
**3**	**10.0**	**8.3**	**4.0**	**4.6**	**0.036**
4	6.3	11.2	2.4	6.2	0.241
**5**	**3.8**	**3.8**	**1.6**	**2.9**	**0.045**
6	1.2	2.4	1.7	3.1	0.571
7	4.9	5.2	6.0	7.7	0.698
**8**	**52.3**	**48.7**	**25.2**	**27.9**	**0.038**
**9**	**24.5**	**26.7**	**9.3**	**14.3**	**0.003**
10	4.5	4.0	4.9	7.8	0.192
**11**	**40.7**	**39.0**	**3.0**	**14.1**	**0.047**
12	11.1	22.7	12.3	21.0	0.733
13	2.0	3.7	2.0	3.0	0.979
14	2.3	3.1	3.2	4.9	0.701

† Top quintile of seizure durations; § Residual quintiles; *Wilcoxon testing, significance determined by FDR level set at 0.15

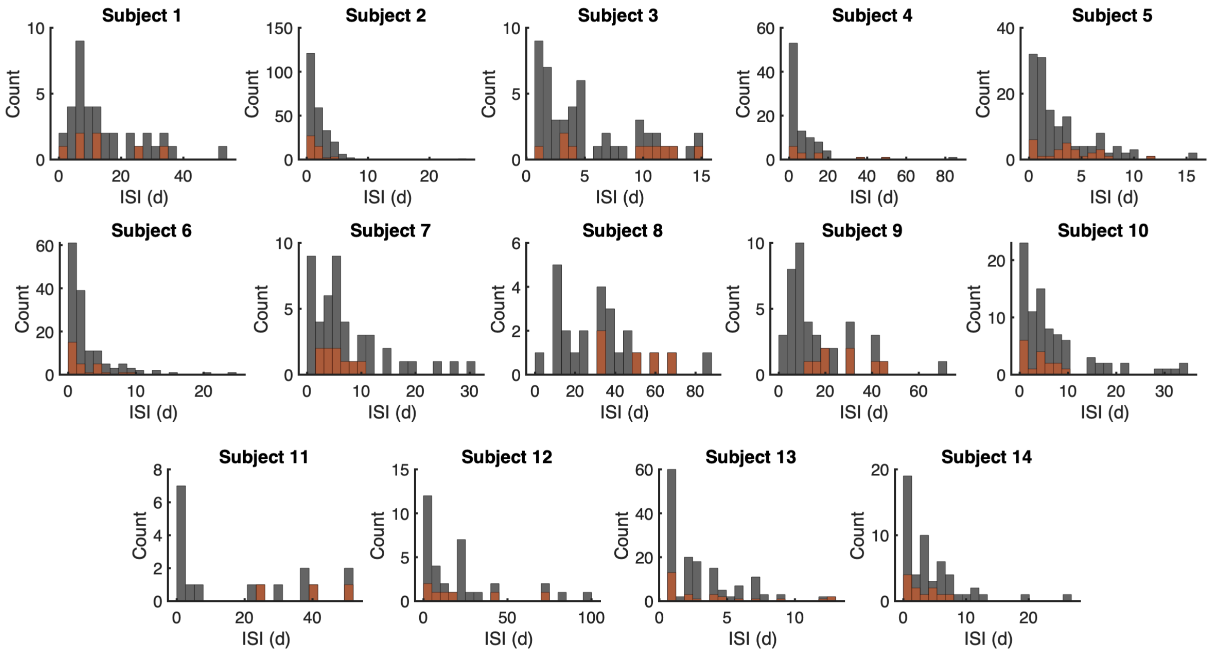

Figure S3. Distributions of ISI for long-duration (red bars, top quintile) and shorter-duration (grey bars, residual distribution) seizures for all subjects.

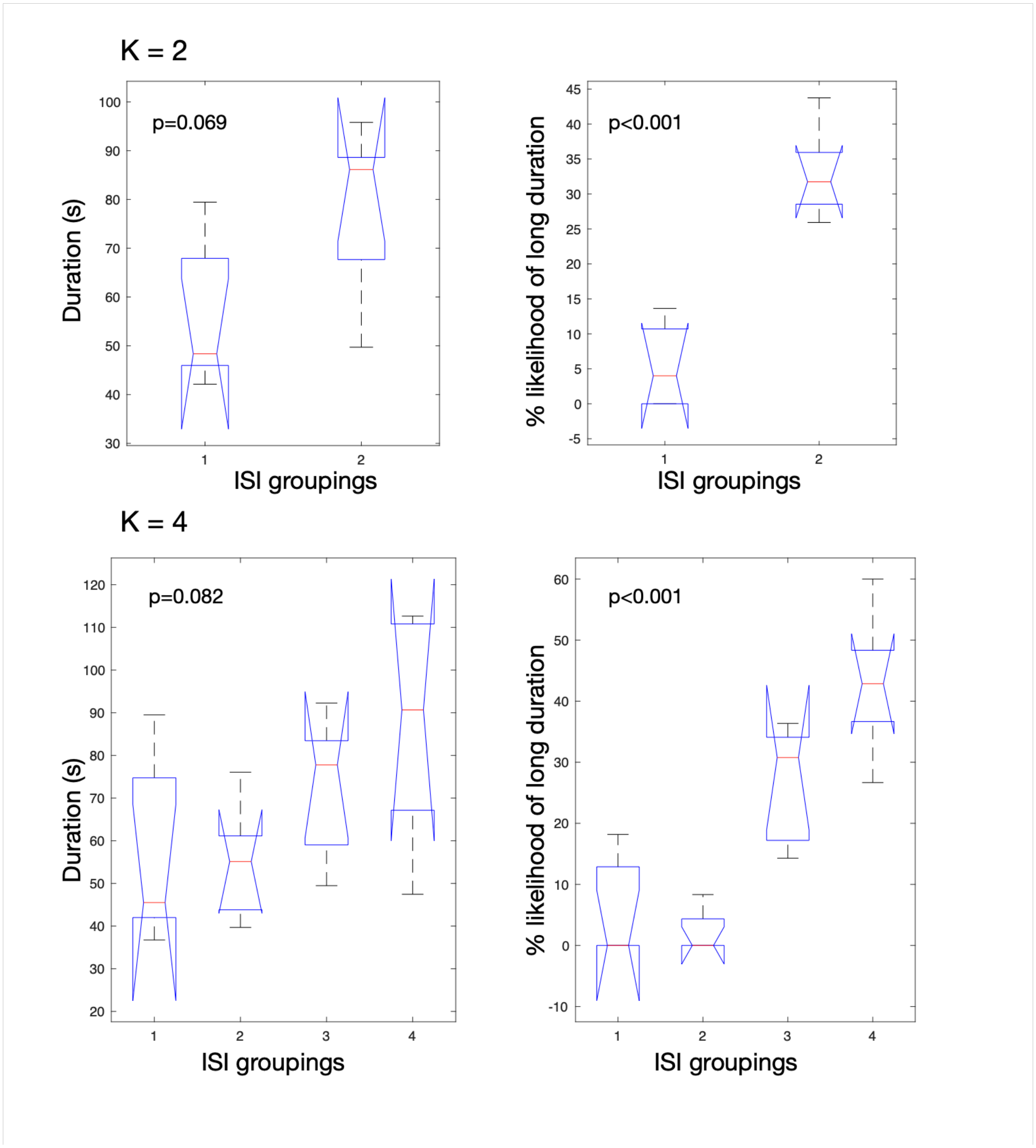

Figure S4. Seizure durations (left) and proportions of long duration seizures (right) across different ISI groupings. The ISI is grouped into K=2 (top) and K=4 (bottom) clusters; see Figure 3A,B for K=3 results. The likelihood of long-duration seizures remains statistically significant by ANOVA testing when considering different number of ISI clusters; however, the seizure durations are not significantly different between the ISI groupings.

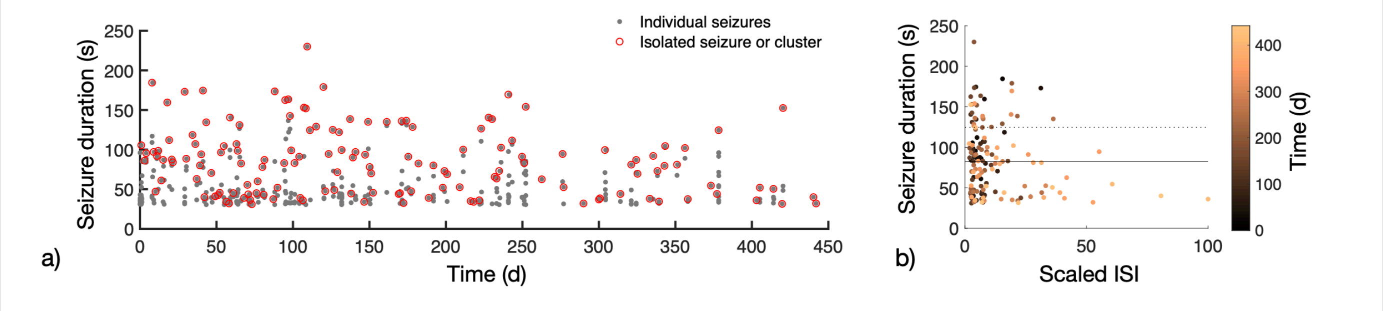

Figure S5: Data from Subject 6 showing A, time series of seizures, and B, seizure duration vs. scaled ISI, colored by time. Seizure control improves over the course of the time series, which is reflected by the longer ISIs and shorter seizure durations (copper) with relatively higher ISI lengths.
